# Re-visiting the call for translation of cancer survivorship research: collaborative multidisciplinary approaches to improve translation and dissemination

**DOI:** 10.1007/s10552-023-01809-5

**Published:** 2023-11-14

**Authors:** Betsy Risendal, Cynthia A. Thomson, Aaron Seaman, Rachel Hirschey, Linda Overholser

**Affiliations:** 1 Colorado School of Public Health, Community and Behavioral Health, University of Colorado Denver, 13001 E. 17Th Place, Bldg 500, MS F538, Aurora, CO 80045 USA; 2 Health Promotion Sciences, University of Arizona, Mel and Enid Zuckerman College of Public Health, Tucson, AZ USA; 3 Department of General Internal Medicine, University of Iowa, Roy J. and Lucille A. Carver College of Medicine, Iowa City, Iowa USA; 4 School of Nursing, University of North Carolina, Chapel Hill, NC USA; 5 School of Medicine Division of General Internal Medicine, University of Colorado Denver, Aurora, CO USA

**Keywords:** Cancer, Cancer survivorship, Dissemination science, Translational science

## Abstract

**Background:**

The number of cancer survivors in the US is dramatically increasing and survivors are living longer, making the ongoing care and quality of life in this growing population an important public health issue. Although there has been significant progress in cancer survivorship research, gaps in translating this research to real-world settings to benefit survivors remain.

**Methods:**

The number and type of cancer survivorship research activities in past and current projects were gathered in reports and work plans from the Cancer Prevention and Control Research Network (CPCRN). Additionally, current cross-center projects were aligned with common constructs in dissemination and implementation science to provide a narrative review of progress on translational research.

**Results:**

A review of historical activities in the CPCRN indicates that there has been consistent engagement in survivorship from multiple institutions over the last decade, generating 84 grants, 168 papers and 162 presentations. The current membership of the Survivorship Workgroup includes multiple disciplines and all 8 participating institutions. Together these Workgroup members have developed 6 projects, all of which address multiple domains in translational research such as feasibility, practicality, and organizational and cultural factors that affect implementation.

**Conclusions:**

This review of past and ongoing activities in the CPCRN suggests that survivorship has been a consistent priority including the translation of evidence-based approaches into practice. Specific gaps in the translational research agenda that could be the focus of future investigations by Workgroup members and others include the practical and logistic aspects of interventions such as cost and policy.

## Background

The number of cancer survivors in the U.S. as of 1 January 2022, is estimated to be nearly 18 million, which represents a 20% increase in less than a decade. This number is rapidly increasing due to the aging of the US population and improvements in early detection and treatment. About half of these individuals will live 10 years or more following a cancer diagnosis, making ongoing care and quality of life issues in this growing population important public health concerns [1]. Efforts by the Centers for Disease Control (CDC), the Livestrong Foundation (formerly the Lance Armstrong Foundation), and others to enhance cancer survivorship research resulted in the National Action Plan for Cancer Survivorship. The goal of this Action Plan was to provide a guide for survivorship research and resource allocation [2]. This collaborative plan was followed by The Institute of Medicine’s landmark report, “Lost in Transition” (2006) [3], which further spotlighted the complex and diverse issues facing cancer survivors. These include increased risk of late and long-term effects of cancer treatment such as cardiovascular disease, depression, and other comorbid conditions, as well as adverse impacts on activities of daily living, psychosocial well-being, and ability to work. Since that time, researchers, clinicians, public health practitioners, and survivor advocates have engaged in extensive efforts to increase our understanding of these issues and study potential solutions.

In 2013, a follow-up study to assess the status of the above Action Plan noted several areas of progress which included education/training, communication, and the gathering and use of surveillance data in survivorship. However, a gap in the uptake and dissemination of research findings to the cancer survivorship community was identified [4]. Recent reports continue to indicate that cancer survivors experience poor health-related quality of life as compared to the general population, especially those who are diagnosed before age 40 or are from racial/ethnic minority groups [5]. Thus, there is a continued need for research findings to be rapidly disseminated and implemented to reach cancer survivors and address these persistent and serious concerns.

Just over a decade ago, Pollack and colleagues issued a call to action to use methods from dissemination and implementation science to move survivorship research findings into real-world settings [6]. Dissemination and implementation science expands traditional research approaches by including research domains of relevance to the translation of findings beyond scientific studies. For example, research translation focuses heavily on: participation and reach (who is not represented, and why?); practical considerations such as feasibility of delivery and adaptability of the intervention; and the relative advantage of the intervention or innovation over current practice. In contrast, traditional research methods focus heavily on tightly controlled settings and restrictive inclusion/exclusion criteria, which limit the usability and applicability of the findings outside of research studies. The application of dissemination and implementation science methods to survivorship requires multi-level approaches that engage partners across roles: research institutions; survivorship health care providers and clinical systems; public health; organizations reaching survivors and community coalitions; and survivors, family members, and friends. The authors put forth this call to action for multi-level translational research to “encourage the conduct of more relevant practice-based research that can improve the care of survivors”.

These conclusions were later supported by Alfano and colleagues in their 2014 report, in which they describe several barriers to translating cancer survivorship research into better care for the growing number of cancer survivors. They cite lack of inclusion of survivors and family members in the research and translation process, which results in findings which may not be relevant or feasible to the survivor; and research “silos” between disciplines which stifle the movement of research findings into practice [7]. The authors propose a full range of research activities from basic science through to implementation science approaches to fully realize the benefits for survivors in real-world settings. This work was contextualized by Nekhlyudov in the 2019 publication of a quality of cancer survivorship care framework that integrated research efforts with care and policy efforts to optimize outcomes in this vulnerable group [8].

The purpose of the current paper is to describe the survivorship research translation activities conducted by members of the Cancer Prevention and Control Research Network (CPCRN) a decade after the publication of initial recommendations. The Cancer Prevention and Control Research Network (CPCRN) is a national network of researchers and practitioners from eight geographically diverse academic centers who work together to address cancer-related disparities. The CPCRN is one of the thematic research networks in the Prevention Research Centers (PRCs) program supported by the Centers for Disease Control (CDC).

To provide context for this report, the CPCRN operationalizes its research efforts through workgroups. Workgroups are tasked with identifying, completing, and disseminating engaged research-based projects. Investigators participate in an in-depth prioritizing process at the beginning of each grant cycle (Cycle 3, 2009; Cycle 4, 2014; Cycle 5, 2019) wherein all participants establish priorities for workgroup formation, and an official voting process is completed to determine the workgroups that will be operationalized for the grant cycle. The workgroup formation process includes an initial academic network evaluation using a network analysis software (KUMU; [9] to identify common research foci and estimate areas wherein a critical mass of investigators is positioned to move a research topic area forward. Once network capacity is described, investigators brainstorm the areas rich for research impact and productivity, and a prioritization process, wherein each is provided a select number of votes to prioritize their interests, is completed. A final vote confirms the workgroups that will be operationalized, generally 3–7 workgroups per grant cycle. Workgroups are charged with developing a workplan, with specified projects and outputs, and timeline for task completion. The workplans undergo final approval by the CPCRN Steering Committee, represented by each funded research site, the Coordinating Center, and the funders. Further, these workgroups are evaluated annually at the investigator meeting in terms of relevance, interest, and priority across emerging topics for cancer prevention and control research.

To our knowledge, this report of the Survivorship Workgroup activities in the CPCRN is the first to provide a longitudinal view of survivorship research in response to the call for implementation science approaches to drive translation of research findings by Pollack et al. The gap identified by Pollack was exemplified in a recent analysis of the survivorship research portfolio at the National Cancer Institute (NCI; [10] wherein the majority (60%) of the nearly 600 funded studies evaluated intervention efficacy, and many of these were pilot or feasibility studies. Of those that indicated an implementation science component (*n* = 23), most were focused on care delivery and similarly studied interventions rather than ways to increase translation. The authors concluded that despite this dearth, the need for implementation science approaches in survivorship is clear. The current report of activities in cancer survivorship research under the CPCRN aims to describe both productivity and engagement in survivorship research translation activities across multiple sites over time. It also addresses an important gap in the literature by describing the implementation science-focused components of current survivorship research activities in the CPCRN, a research network with national scope, in order to provide insights on progress toward improving the translation of research findings to practice.

## Methods

This report evaluates the research activities conducted by the Cancer Survivorship Workgroup in the CPCRN, and individual sites, as described in annual reports and workplans from the Network Coordinator Center using the key word “survivor”. The evaluation included a review of previous cycles (Cycle 3, 2009–2014 and Cycle 4, 2014–2019) through to recently completed and current projects conducted in Cycle 5 of the CPCRN (2019–2022; years 1–2.5 of the 5-year grant cycle). The information from Cycles 1 and 2 were not recorded and categorized in the same way Cycles 3–5, and therefore are not included in the current report.

In a separate examination, projects in the current Cycle 5 Survivorship Workgroup were evaluated for their use of multi-level implementation science approaches to support research translation. The Workgroup projects are led by numerous subgroups focused on specific topics which include nutrition and physical activity, special populations (adolescent and young adult, geriatric cancer patients), COVID-19 and cancer, and health equity (see also Results, Table). Activities in these subgroups in the current cycle (Cycle 5) were mapped to factors identified by Pollack et al. [6] to describe how workgroup activities address research translation. Specifically, Pollack et al. recommended the use of the well-established RE-AIM Framework (Reach, Effectiveness, Adoption, Implementation and Maintenance; [11]) and the K2A Framework (Knowledge to Action; [12]) to identify factors relevant to the translation of survivorship research into practice.

**Fig. 1:**
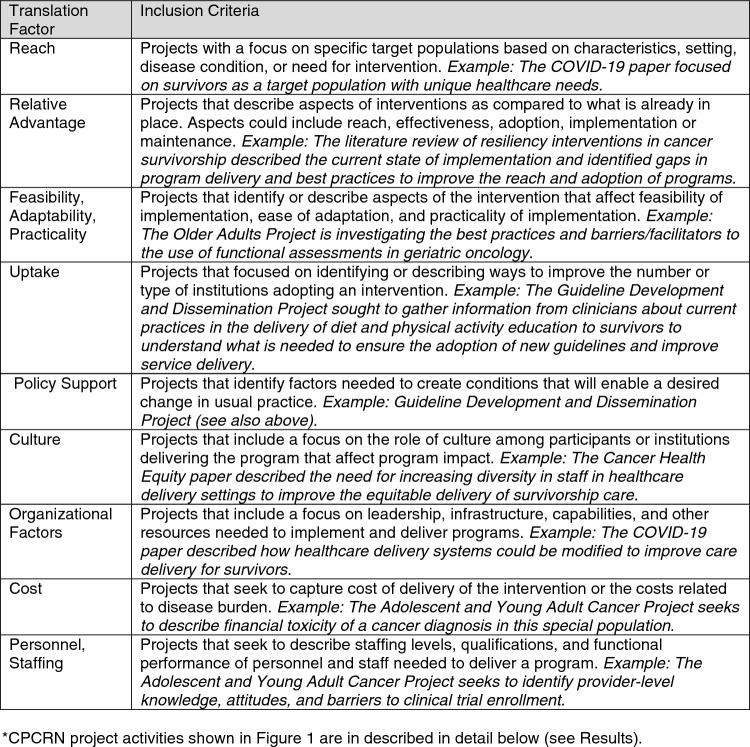
Translation Factors and Inclusion Criteria for Mapping of CPCRN Project Activities*

Important translation factors, identified by these frameworks, guided this evaluation of CPCRN survivorship research. As described by Pollack et al., factors from these frameworks relevant to translation to practice include but are not limited to: reach, relative advantage, feasibility, adaptability, practicality of implementation, uptake, policy support, culture (in the organization and the target population), organizational factors, cost, and personnel/staffing. Definitions of each that guided the mapping are shown in Fig. 1.

Pollack et al. also define multi-level partners who are essential to the translation of cancer survivorship research, partners who were integrated into Survivorship Workgroup efforts. These include research institutions, public health, organizations, communities (providers, coalitions), and individuals (survivors, caregivers, family members; see Table 3).

A single investigator mapped the projects to both the implementation science factors and the multi-level partners based on a review of meeting minutes, project workplans, and annual reports collected through routine Workgroup operations. The analysis of annual reports was provided by the Network Coordinating Center. Meeting minutes and project workplans were generated in the current grant cycle, and maintained and distributed each month to all members including the authors. Meeting minutes included descriptions and progress related to project activities which were used to identify components relevant to translation. The co-authors of the current report reviewed and confirmed these results based on their in-depth knowledge of the projects through regular participation in subgroup and Workgroup activities.

## Results

Research-related outputs including those focused on translation of findings into practice using implementation science approaches are shown in Table 1, below. Survivorship-related activities over the last decade have generated 84 grants, 168 papers and 162 presentations. Notably, the last category shown in Table 1 (reports, plans, policies and guidelines) is where academic expertise and outputs (e.g., publications and presentations) are translated into products with broader applicability and reach. The engagement of CPCRN members in the development of state cancer plans, as well as survivorship care guidelines issued by the National Comprehensive Care Network (NCCN) and the American Cancer Society (ACS), represent examples of the Network members’ contributions to these broader translation products. Important to this report, cancer survivorship was identified as a priority in all cycles; however, in the 2014–2019 Cycle 4, the decision was made to de-centralize cancer survivorship activities across workgroups rather than have a specific workgroup effort. The information on Cycle 5 reflects activities up to the mid-point in the grant cycle.

**Table 1:** CPCRN cancer survivorship translational research outputs over time (2009–2022), including activities at individual sites and in workgroups

	Cycle 3 10–2009 to 09–2014 (sites, out of 9)	Cycle 4* 10–2014 to 09–2019 (sites, out of 8)	Cycle 5 Mid-point 10–2019-09–2022 (sites, out of 8)
Submitted grants	55 (8 sites)	19 (7 sites)	10 (6 sites)
Publications	45 (8 sites) *	65 (8 sites)	58 (8 sites)
Presentations	111 (6 sites)	33 (6 sites)	18 (6 sites)
Reports, plans, policies, guidelines	15 (5 sites)	8 (6 sites)	4 (3 sites)

*Cycle 4 includes research outputs from workgroups other than Cancer Survivorship as cancer survivorship efforts were decentralized across all CPCRN workgroups during this funding cycle

### Translational activities in the current survivorship workgroup

Workgroup membership in the current cycle includes over 70 individuals representing all 8 Network sites plus affiliate members at other institutions with multiple grant roles at their respective performance sites (Co-Is, PIs, research coordinators, and trainees). Disciplines represented include epidemiology, communication science, public health, nutrition, nursing, primary care, and health behavior/psychology, among others. Cancer survivors and government employees are also represented. The Workgroup meets monthly, and agendas regularly include health equity, anti-racism and patient-focused readings and resources which are discussed as a group. The current and completed projects at the mid-point in Cycle 5 are described below:

### Guideline development and dissemination (American cancer society nutrition and physical activity guide for cancer survivors (*n* = 10 members)

This project includes contributions to the development of updated cancer survivor guidance and efforts in dissemination of the guidelines to healthcare providers. The guideline has been published and the dissemination efforts aim to conduct a survey of healthcare providers to evaluate current knowledge, attitudes, and provider training practices regarding the American Cancer Society (ACS) Nutrition and Physical Activity Guidelines for Cancer Survivors [13, 14]. The goal of the survey is to understand how to support the implementation and dissemination of these evidence-based guidelines and improve patient care. Questions in the survey include items regarding program reach, knowledge and attitudes about guidelines (relative advantage), current practices and beliefs about uptake (feasibility, adaptability and practicality), facilitators and barriers of guideline uptake, and influence of various factors on uptake (policy, culture, organizational factors such as role of staff). Presentation of the guideline at relevant professional meetings (e.g., ASPO, FNCE, National Comprehensive Cancer Coalition, etc.) is another activity under this subgroup (reach).

### Special populations—adolescent and young adult (AYA) cancer survivors

This subgroup (*n* = 5 members) developed a survey to understand perceived clinical provider-level barriers and facilitators of the recruitment of AYA survivors in clinical trials. Similar to the above survey, this tool addresses the unique characteristics of AYA survivors and their providers (reach), knowledge and attitudes about clinical trials (relative advantage), current practices and beliefs about uptake (feasibility, adaptability and practicality), facilitators and barriers of uptake (organizational factors, cost). Additionally, a qualitative study is in development to explore issues of financial toxicity for young adults with a history of childhood cancer who have recently transitioned to an adult health care setting. This project examines specific practical concerns for a population with unique health care needs (reach, and cost).

### Special populations—older adults

This 14-member subgroup is conducting a qualitative study with oncology and primary care clinicians to investigate the use of functional assessments in geriatric oncology settings to understand how assessments are used in clinical practice. The goal is to identify best practices and barriers/facilitators to implementation. Geriatric assessments are key to assessing potential treatment toxicities, treatment options, and potential supportive care needs yet are not widely implemented. Questions in the survey include items regarding program reach, knowledge and attitudes about guidelines (relative advantage), current practices and beliefs about uptake (feasibility, adaptability and practicality), facilitators and barriers of guideline uptake, and influence of various factors on uptake such as organizational factors and role of staff).

### Resiliency in cancer survivorship (*n* = 5 members)

The goal of this subgroup is to identify best practices in the design, implementation and dissemination of resiliency interventions by conducting a systematic review of interventions among caregivers of childhood cancer patients, a highly distressed group (completed; to be submitted for publication). Best practices for implementation and maintenance of resiliency interventions identified in this review will be discussed by the Workgroup to extend findings to other survivors as well as the general population in response to the growing mental health and coping issues associated with the COVID-19 pandemic. Items abstracted for the review included those related to perceived effectiveness in real-world settings as compared to usual care (relative advantage), the role of of culture, and staffing needs for implementation.

### Meeting immediate needs—COVID-19 and Cancer Survivorship (completed; *n* = 10 members)

The Survivorship Workgroup described the adaptation of the Cancer Survivorship Quality Care Framework under COVID [15] and use of public health approaches to help ensure existing disparities in survivorship are not magnified as a result of the pandemic [16]. Collectively these papers address reach by describing unique challenges in survivorship; barriers and facilitators to implementation (feasibility, adaptability, practicality); factors that impact the delivery of public health interventions in clinical settings (organizational factors); and the delivery of messaging and care to survivors (personnel/staff).

### Cancer health equity in survivorship care (*n* = 9 members)

The goal of this collaborative work is to understand the influence of racism on equitable survivorship care, using health equity principles developed by the CPCRN Health Equity workgroup. Recommendations to strengthen CPCRN capacity for conducting heath equity research are provided and guiding current CPCRN practices. [17]. Of note, these efforts also informed the successful ACS funding of a new Cancer Health Equity Research Center at one of the active CPCRN research sites. The development of these recommendations addresses several relevant implementation-related factors including reach and culture (encouraging representation of diverse investigators and survivors in research through community advisory boards and other activities); the importance of improving the delivery of quality care for all (relative advantage; uptake); and barriers and facilitators of equitable survivorship care with a focus on care team diversity in composition and infrastructure (organizational and staff factors).

A summary of the narrative evaluation of these activities conducted by current Survivorship Workgroup members (2019–2022) according to domains in the survivorship translation and dissemination research agenda set out by Pollack et al. in 2011 is shown in Table 2 (below). In sum, these data support a focus on diverse domains and suggest the potential to impact cancer survivors through translational research efforts is being realized.

**Table 2:** Mapping of current CPCRN cycle 5 survivorship workgroup projects* to domains in practice-based research and translation cancer survivorship research

	Guideline development and dissemination	Special populations: adolescent and young adults	Special populations: older adults	Resiliency in cancer survivorship	Meeting immediate needs: COVID-19 and cancer survivorship	Cancer health equity in survivorship care
Reach, targeted population	X	X	X		X	X
Relative advantage**	X		X	X		X
Adaptability, feasibility, practicality	X	X	X		X	
Uptake	X	X	X			X
Policy support	X					
Culture	X	X	X	X		X
Organizational factors	X		X		X	X
Cost		X				
Personnel, staffing	X		X	X	X	X

*Workgroup projects from 2020 to 2022 only

**Relative advantage is defined in Roger’s diffusion of innovation as the degree to which an innovation is seen as better than the idea, program, approach, or product it is intended to replace [6]

The 2011 call to action by Pollack et al. explicitly described the need for multi-level partnerships and dissemination efforts to advance cancer survivorship translational research efforts and impact. Table 3 details the diversity and level of partnership engagement according to current individual Survivorship Workgroup activities. By nature, the CPCRN is academia-based, thus research partnerships are inherent in every activity. Beyond academia, we see frequent engagement of healthcare providers, comprehensive cancer centers, and cancer survivors. Public health partnerships are in the forefront of broader public health activities such as addressing the impact of the COVID pandemic on cancer survivorship and cancer health equity.

**Table 3:** Multi-level Approaches in CPCRN Cycle 5 Survivorship Workgroup Projects

	Guideline development and dissemination	Special populations: adolescent and young adults	Special populations: older adults	Resiliency in cancer survivorship	Meeting immediate needs: COVID-19 and cancer survivorship	Cancer health equity in survivorship care
Research institutions (NIH, Academia)	X	X	X	X	X	X
Public health partners (CDC, schools of public health, policy)					X	X
Organizations (service to survivors, professional)	X		X		X	X
Community (healthcare, comprehensive cancer centers)	X	X	X	X	X	X
Individuals (e.g., survivors, family members, caregivers)	X	X			X	X

## Discussion

CPCRN has been productive in the survivorship arena for well over a decade. In the decade following the 2011 call to action for translational survivorship research [6], it is apparent that multiple investigators in leading cancer centers participating in the CPCRN Survivorship Workgroup have decidedly moved in this direction. As evidenced in Table 2, projects address all domains relevant to research translation and many projects span multiple domains. Projects that include a focus on reach, feasibility, and uptake were common, in keeping with the Network’s focus on dissemination and implementation. A focus on cultural aspects of implementation were identified across all projects, aligned with the CPCRN identify toward cancer health equity in programming. Importantly, opportunities highlighted in the paper by Pollack [6] to conduct projects with a multi-level and researcher-practitioner focus are apparent in the latest grant cycle, with a notable emphasis on cross-site projects engaging researchers with healthcare providers and survivors (Table 3).

One possible explanation for these positive findings is that the CPCRN Survivorship Workgroup structure directly addresses at least one of the two major barriers noted by Alfano et al. [7], which is the barrier created by research “silos”. As previously noted, the Survivorship Workgroup includes members from multiple disciplines thus promoting the exchange of ideas and approaches. Workgroup activities engage diversity in membership including survivors, clinicians, representatives of public health organizations and organizations servicing the needs of cancer survivors, strengthening the voice that informs on the research in terms of translational potential and impact.

A barrier addressed by the Cancer Survivorship Workgroup and noted by Alfano et al. is regarding the lack of inclusion of the survivor voice in the translation process. This has been addressed in part by the Workgroup structure and membership, which includes the survivor voice. Many Survivorship Workgroup members are caregivers, cancer survivors, or clinicians who provide care for survivors (including oncology, primary care and behavioral health). Additionally, the agendas at the monthly meetings are structured to include patient-centered readings and group discussions.

The decline in overall numbers related to outputs in cancer survivorship from Cycle 3 to the subsequent cycles may suggest concern; however, an assessment of changes in CPCRN operations, documentation and the broader cancer survivorship research environment lends insight on these numbers. First, the large number of presentations and reports during Cycle 3 likely represents a period when there were multiple survivorship conferences and guidelines being developed throughout the U.S. notably the Biennial Cancer Survivorship Meeting and Livestrong Centers of Excellence. During Cycle 4, several of these national opportunities and efforts for dissemination ceased. The number of presentations also decreased over time suggesting that it may be more challenging to find appropriate, visible venues translational cancer survivorship research as more novel areas of scientific inquiry emerge (e.g., Artificial intelligence, precision prevention and therapeutics, etc.). Interestingly, during Cycle 4 the cancer survivorship specific WG was not identified for continuation, and rather CPCRN-related cancer survivorship efforts were distributed over all workgroups. In 2020, the Cancer Survivorship Workgroup was reinstituted (Cycle 5) to again prioritize cancer survivorship efforts. Unfortunately, the Workgroup activities were somewhat curtailed by the COVID pandemic and related survivorship activities were required to shift, arresting several ongoing efforts. The WG actively addressed the changing survivorship environment and developed a workplan to enhance efforts with consideration for the pandemic environment [15, 16].

When the original article calling for translational research was published in 2011 [6], there was a relatively large amount of Survivorship Workgroup centric activity particularly in terms of grant submissions (Cycle 3), suggesting that this was a time of hypothesis testing (efficacy) and generating emerging evidence (T1-T2 translation) that prompted more T3-T4 translational efforts over time. Cycle 5 is on track to be very productive especially with dissemination outputs such as papers and presentations and even guidelines and policy, suggesting work focused in the T3-T4 translation continuum. Exemplar of this T3-T4 emphasis in our work, WG members have engaged in ACS guideline development for nutrition and physical activity in cancer prevention [12] and cancer survivorship [13] as well as new guidance for diet and physical activity during cancer treatment [18]. In addition, the COVID Cancer Survivorship papers informed changes in cancer survivorship quality care necessitated by the competing needs of cancer survivors during an active pandemic [15, 16].

The insights provided in this paper add to the importance of the issues raised a decade earlier by demonstrating that progress is being made. However, there are still opportunities to fill gaps in translational research to directly benefit cancer survivors. For example, this narrative review of current and recently completed CPCRN Survivorship Workgroup projects and related outputs indicates that relatively few are addressing practical issues relevant to implementation such as cost and policy supports, which are key factors for the dissemination of evidence into practice. As the evidence base around cancer survivorship care has grown over the last decade and clinical practice guidelines are now available from multiple clinical care organizations, it will be necessary to understand how to best disseminate and implement these guidelines in individual practice settings. Although many projects have a patient-centered approach, greater application of community-based participatory research (CBPR) methods, as is central to dissemination and implementation science, should afford greater impact over time. Future efforts should account for the time and complexity of using CBPR methods in cross-site projects in practice-based survivorship research. Finally, while most projects engage public health agencies, community organizations and / or health care institutions in project development and implementation, this will continue to represent another area of opportunity not fully explored in recent Workgroup activities. The growing number of multidisciplinary researchers interested in cancer survivorship with expertise in dissemination and implementation science as exemplified in the CPCRN is an asset toward filling these gaps. As identified in a recent workshop by the National Academy of Sciences, engagement of multidisciplinary care team members in cancer survivorship and related care-based research will afford additional opportunities to integrate dissemination and implementation science resulting in more effective survivorship care delivery [19].

The strengths of this narrative evaluation of translational emphasis of CPCRN Cancer Survivorship Workgroup efforts are the ability to assess the Workgroup and broader CPCRN efforts in translational cancer survivorship research productivity over time. Further, we were able to map progress against the domains of activity described by Pollack just over a decade ago and to track progress in relation to shifts in the broader field of cancer survivorship translation and dissemination science, thus illuminating areas for future research.

Limitations of the narrative evaluation relate to the retrospective evaluation of research activities/ projects and outputs based on a key word search, not having established an a priori approach to this evaluation that would likely have provided a more comprehensive assessment. For example, within the workgroup focused on cancer prevention and control, implementation of evidence-based screening strategies is a priority, and this effort includes promotion of screening among cancer survivors residing in rural communities.

The efforts among the subgroup members featured in Tables 2 and 3 are ongoing at this point in the grant cycle, limiting our ability to describe the impact of these approaches. The primary source material (e.g., meeting minutes) did not quantify the descriptive information reported in these tables; however these quantified data are key to monitoring progress in the survivorship research agenda and should be collected in future investigations. An important limitation to note is that the narrative summaries of multi-level implementation science approaches shown in Tables 2 and 3 do not represent all peer-reviewed cancer survivorship research being conducted at institutions in the CPCRN and reflect only that of the Survivorship Workgroup. A more comprehensive and rigorous review of translational research in the field to confirm our findings is needed.

## Conclusion

In their report of the 25-year history of the Office of Cancer Survivorship (2021) [20], the National Cancer Institute recommended continued emphasis on the integration of implementation science strategies into the field of survivorship research. The current overview of survivorship-related research in the CPCRN, which was guided by the call to action issued by Pollack et al. [6], suggests that the translation and dissemination of cancer survivorship research to real communities and practice-based settings remains a priority and is the focus of ongoing activities. The call for multi-level research with an increasing focus on healthcare delivery and system level issues as described by the CDC in the Public Health Action Model for Cancer Survivorship [21] is also evident across Workgroup projects. This review also offered insights into specific gaps in the translational research agenda that could be the focus of future investigations by Workgroup members and others, including those related to cost and policy.

The CPCRN Survivorship Workgroup may be a ‘bellwether’ for cancer survivorship research with a translational focus, given that the focus of the Network at large is to promote the translation and dissemination of evidence-based approaches. Additionally, the representation of diverse geographic regions and high-performing academic institutions across the US who are awarded the funding to participate in the Network further supports the notion that CPCRN investigators are likely on the leading edge of research trends. Given the broad representation of institutions, partners and disciplines represented in the Workgroup, it is likely that new knowledge and expertise for practice-based and translational research is being transferred beyond Workgroup projects, resulting in broader impact on the field. The types of activities conducted by members of the CPCRN have the potential to advance understanding of the multi-level factors and partners important in the care of cancer survivors and improve the design, testing and dissemination of evidence-based interventions to meet the needs of this growing population.

## Data Availability

Not applicable.
